# The relation between meniscal dynamics and tibiofemoral kinematics

**DOI:** 10.1038/s41598-024-59265-3

**Published:** 2024-04-17

**Authors:** A. Van Oevelen, M. Peiffer, A. Chevalier, J. Victor, G. Steenackers, E. Audenaert, K. Duquesne

**Affiliations:** 1 Department of Orthopedic Surgery and Traumatology, Ghent University Hospital, Corneel Heymanslaan 10, 9000 Ghent, Belgium; 2 Department of Human Structure and Repair, Ghent University, Corneel Heymanslaan 10, 9000 Ghent, Belgium; 3 Department of Electromechanics, InViLab research group, University of Antwerp, Groenenborgerlaan 171, 2020 Antwerp, Belgium; 4 Cosys-Lab Research Group, Department of Electromechanics, University of Antwerp, Antwerp, Belgium; 5 Department of Trauma and Orthopedics, Addenbrooke’s Hospital, Cambridge University Hospitals NHS Foundation Trust, Hills Road, Cambridge, CB2 0QQ UK; 6 imec-VisionLab, Department of Physics, University of Antwerp, Universiteitsplein 1, 2610 Wilrijk, Belgium

**Keywords:** Anatomy, Biomedical engineering

## Abstract

Over the past 30 years, research on meniscal kinematics has been limited by challenges such as low-resolution imaging and capturing continuous motion from static data. This study aimed to develop a computational knee model that overcomes these limitations and enables the continuous assessment of meniscal dynamics. A high-resolution MRI dataset (n = 11) was acquired in 4 configurations of knee flexion. In each configuration, the menisci were modeled based on the underlying osseous anatomy. Principal Polynomial Shape Analysis (PPSA) was employed for continuous meniscal modeling. Maximal medial anterior horn displacement occurred in 60° of flexion, equaling 6.24 mm posteromedial, while the posterior horn remained relatively stable. At 90° of flexion, the lateral anterior and posterior horn displaced posteromedially, amounting 5.70 mm and 6.51 mm respectively. The maximal observed Average Surface Distance (ASD) equaled 0.70 mm for lateral meniscal modeling in 90° of flexion. Based on our results, a strong relation between meniscal dynamics and tibiofemoral kinematics was confirmed. Expanding on static meniscal modeling and employing PPSA, we derived and validated a standardized and systematic methodological workflow.

## Introduction

Taking more than 1 million steps in bipedal gait every year, the knee joint is subjected to a significant cumulative mechanical stress^1^. Due to the crucial role of the menisci in distributing pressure over the tibial plateau, researchers have been captivated by the dynamics of the meniscus for over 30 years now^2,3^. The historical paper by Thompson and colleagues, published in the early nineties, was the first to elucidate meniscal kinematics in an intact cadaveric knee, and their findings continue to serve as the benchmark in understanding meniscal dynamics today^2^.

Thompson and colleagues utilized Magnetic Resonance Imaging (MRI) to visualize meniscal movement without invasive dissection of the joint. They mounted a cadaveric knee on a mobile crossbar system and scanned it throughout the complete range of flexion–extension in 10° increments. Their research revealed that as knee flexion increased, the menisci adapted to maintain contact with the femoral and tibial articular surfaces, thereby improving joint congruence^2^.

Although Thompson and colleagues paved the way for studying meniscal dynamics in intact knees, their methodology had significant limitations inherent to the imaging and computational capabilities of that time. First, they were constrained by the resolution and magnetic field strength of the MRI infrastructure, which had an impact on the quality of manual segmentations of the 3D meniscal geometry^2,4^. Second, their observations were conducted on cadaveric knees, which lacked a functional soft tissue envelope that could have influenced tibiofemoral kinematics and meniscal movement^2,5^. Lastly, their methodological set-up did not allow for exact determination of the osseous angle, making it challenging to compare meniscal position at precise degrees of knee flexion between subjects.

Since the groundbreaking paper of Thompson, only a limited number of authors have successfully replicated the experiment in vivo, thereby addressing the limitations associated with cadaveric studies^5–15^. Specifically, a select few authors have been able to perform knee scans in deep flexion, exceeding 60°, utilizing either open or a compact MRI scanner^5,7,8,12,14,15^. Relying on the prevailing techniques available at the time, the majority of studies have been conducted using MRI scanners with low magnetic field strength^5–7,9,10,13–15^. While Yamamoto et al. employed a 2.0 Tesla compact MRI enabling full knee flexion imaging, their scans were acquired in non-weight-bearing conditions^12^. Liu et al. improved upon the magnetic field strength, utilizing a 3 Tesla MRI, but solely captured the loaded knee between 0° and 30° of flexion^11^. Furthermore, there currently exists no comprehensive methodology for accurately evaluating 3D meniscal displacement and anatomical deformations^2,7–14^.

To address these limitations, we have incorporated the latest advancements in 3D technology and computational modeling to develop a personalized and dynamic morphological knee model, inspired by the work of Van Oevelen and colleagues. In this model, the meniscal geometry is represented as a deformable, volume-preserving mesh that conforms to the underlying osseous morphology^16^. By utilizing this approach, we can bypass the time-consuming manual segmentation process and obtain a straightforward three-dimensional analysis of the menisci. To improve upon the discrete evaluation of meniscal dynamics described by Thompson and colleagues, we employ Principal Polynomial Shape Analysis (PPSA) for continuous characterization of meniscal movement. PPSA, recently introduced by Duquesne and colleagues, facilitates statistical shape modeling of non-linear shapes^17^. This continuous description of meniscal position not only allows us to identify cases with equivalent degrees of flexion but also enables standardization for meaningful comparisons.

The primary aim in developing the dynamic, elastic meniscal model is to investigate the relation between meniscal kinematics and the kinematics and morphology of the tibiofemoral joint. This enables us to predict meniscal behavior at any degree of knee flexion, with the menisci conforming to the femoral condyles. Integrating multiple innovative computational tools, our study aims to achieve the following objectives: (1) establish a methodological setup for in vivo imaging of deep knee flexion using a 3 Tesla MRI, (2) create a dynamic, elastic knee model based on the theory that the meniscus conforms to the underlying bony anatomy, (3) validate the newly developed model against manual segmentations, (4) continuously model meniscal dynamics between 0° and 90° of knee flexion, and (5) develop a straightforward measurement technique for assessing the meniscal displacement taking advantage of correspondence and statistical shape models. Our comprehensive approach seeks to provide improved insights into meniscal movement while minimizing the need for extensive imaging or invasive dissection.

## Material and methods

### Data collection

A total of 12 participants, consisting of 6 males and 6 females, aged between 18 and 30 years, were included in the study. These individuals had no history of intra-articular knee pathology or lower limb fractures, and they were free from any lower-limb complaints at the time of MRI scanning. To ensure adequate knee flexion during the MRI procedure, the height of the included participants was limited to 1.70 m. All volunteers were of European descent. Out of the 12 cases, one case was excluded from further analysis as a discoid meniscus was identified during data processing. The mean age, length and weight of the included cases (n = 11) equaled 25.4 years (SD 2.58), 1.65 m (SD 0.07) and 65.3 kg (SD 14.11) respectively.

A high-resolution dataset was acquired using a Vantage Galan 3 Tesla Canon® MRI machine with a 71 cm bore to assess the kinematics of the menisci and for validation purposes. A Proton Density (PD) weighted MRI sequence was used to visualize the menisci and the bony contours. For the dedicated knee scans, a pixel size of 0.3571 mm to 0.3571 mm and a slice thickness of 1.5 mm was obtained. The coronal overview scans had a pixel size of 0.7308 mm to 0.7308 mm and a slice thickness of 3 mm.

**Figure 1:**
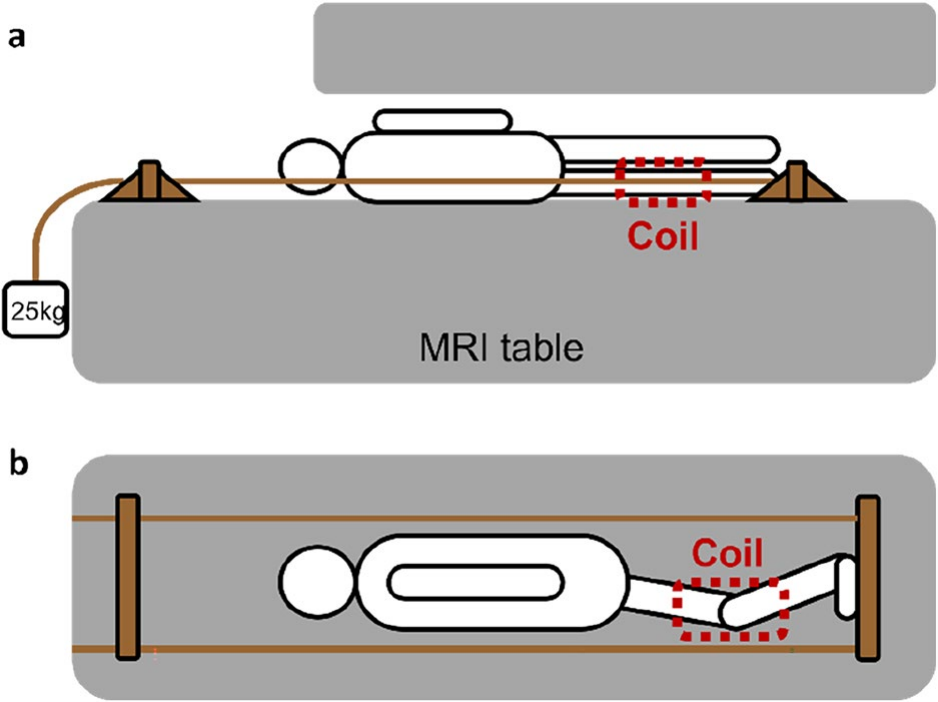
Experimental set up; (**a**) side view and (**b**) top view. The subject was positioned in lateral decubitus on the MRI table, with the foot of the dominant leg placed against a wooden plank. To load the dominant leg, a 25kg weight was attached to the plank using a cable. A coil was placed around the dominant leg for scanning purposes. The non-dominant leg was positioned to avoid any interference. A total of five scans were conducted: four scans were taken with the dominant leg loaded at angles of 90°, 60°, 30°, and 0° of knee flexion, and finally, an unloaded full lower limb scan was performed with the dominant leg at 0°.

Dedicated three-dimensional knee scans were conducted with the knee in different configurations of flexion, while the participant positioned themselves in lateral decubitus on the MRI table with the foot of the dominant leg pressed against a wooden bar. A weight of 25 kg was attached to the latter to simulate knee joint loading in lateral decubitus. The degree of knee flexion was approximated using a goniometer and started at 90°. This setup was repeated for 60°, 30°, and 0° of knee flexion, as illustrated in Fig. 1. Additionally, an unloaded coronal overview scan was conducted to map the lower limb from the hip to the ankle joint. This required 3 separate scan blocks, taken in 0° knee flexion after weight removal. These 3 scan blocks were stitched using the Canon® MRI software, in the formation of an overview, lower limb scan.

The study was conducted in accordance with the Declaration of Helsinki and the Guidelines for Good Clinical Practice. A written informed consent was obtained from all participants, and the investigation was approved by the ethical committee of Ghent University Hospital (IRB B6702021000905).

### MRI segmentation

All MRI scan data was exported as Digital Imaging and Communications in Medicine (DICOM) files and then imported into Materialise’s Interactive Medical Image Control System (Mimics^®^ v21.0, Materialise, Leuven, Belgium). The subsequent calculations were carried out in Matlab^®^ (version R2021b, MathWorks, Natick, MA, USA) using both the Matlab^®^ plugin in the Mimics software and custom-made Matlab^®^ scripts.

#### Segmented osseous anatomy

In the loaded 0°, 30°, 60° and 90° degrees of knee flexion set-up, only the distal femur and proximal tibia-fibula were scanned. However, in the unloaded case at 0° of knee flexion, the full lower dominant limb was scanned, allowing for the acquisition of the entire femur and tibia-fibula complex.

For the unloaded case, the osseous anatomy was derived using a semi-automated segmentation method, as previously described by Audenaert and colleagues^18^. The cortical edges of the femoral and the combined tibia-fibular bone were identified by fitting validated femoral and tibia-fibular statistical shape models (SSMs) to match the cortical edges^18–20^. The retained 25 principal components in the SSM fitting accounted for cumulative explained variances of 99.15% for the femoral bone and 98.43% for the combined tibia-fibular bone. As a result, the femoral and tibia-fibular structures in the overview, lower limb scans were described three-dimensionally as triangulated meshes with an average edge length of 1.77 mm and 1.24 mm respectively^18^. The three-dimensional femoral and combined tibia-fibular bony structures were further used in segmentation of the dedicated knee scans.

**Figure 2:**
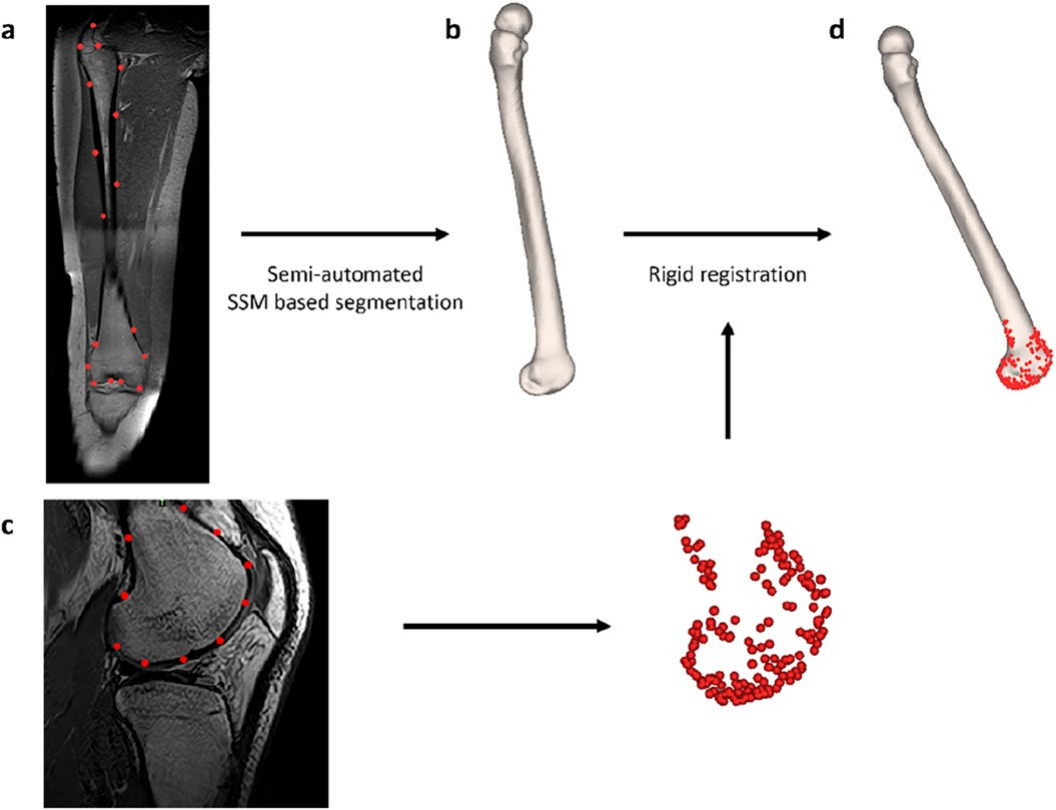
Methodological workflow for semi-automated segmentation of the femoral bone. (**a**) 300 points were randomly selected over the outer surface of the femoral bone on the overview scan. (**b**) SSM-based segmentation was used for the development of a three-dimensional femoral bone. (**c**) Manual allocations on the 30° dedicated knee scan resulted in a point cloud that covered the femoral cortex. This point cloud enabled rigid transformation of the femoral mesh, originating from the overview scan, to match the osseous edges. (**d**) the initial three-dimensional femoral bone was positioned according to the point cloud. The same workflow was applied for 60° and 90° of knee flexion and subsequently for segmentation of the tibial bone.

To determine the exact position of the bones in the loaded cases a Procrustes transformation was employed. The osseous meshes previously obtained from the overview scan were rigidly transformed towards the cortical edges of the distal femoral bone and proximal tibia-fibular bone. This transformation allowed for accurate alignment of the osseous anatomy with the manually determined landmarks on the dedicated knee scans. While preserving the shape, the femoral bone was positioned based on the corresponding MR images, as illustrated in Fig. 2.

#### Segmented meniscal anatomy for validation purposes

For every subject and dedicated scan, manual segmentation of both the medial and lateral meniscus was performed using the Mimics^®^ software^16^. The segmentation process involved meticulous determination of the meniscal edges, aided by the built-in Livewire function. This interactive tool assisted in accurately tracing the contours of the menisci. Segmentation masks were subsequently converted into three-dimensional volumes of the medial and lateral menisci, providing a comprehensive representation of their anatomical structures.

### Subject-specific, soft tissue modeling

#### Cartilage anatomy prediction

Van Oevelen and colleagues have previously developed a method to predict the cartilage layers of the tibiofemoral joint using mean cartilage thickness maps. In this method, the mean cartilage thickness was scaled based on the length of the femur and then applied to the corresponding articular surface to achieve subject-specific predictions. By establishing correspondence between the osseous meshes, the localization of the cartilage layer could be generalized to any new shape^16^.

Consequently, the subject-specific and node-dependent cartilage thickness could be easily obtained and applied to the tibiofemoral joint at different degrees of knee flexion along the surface normal. This approach enabled the modeling of realistic cartilage thickness variations specific to each subject.

#### Meniscal anatomy prediction

Van Oevelen and colleagues modeled the menisci as elastic, volume preserving structures accommodating to the shape of the femoral condyles in the extended knee. The menisci were attached to the tibial plateau at the anterior to the posterior horn. These attachments were initially defined based on MRI data. Additionally, MRI measurements were used to determine the varying height and width over the meniscal course. These measurements informed the creation of an initial tube with variable dimensions. Different triangles were then formed from the initial tube to represent the meniscal shape. In the final optimization step, the edges of triangles penetrating cartilage or bone were adjusted to accurately reflect the femoral condyle geometry^16^.

Here, this methodology was adopted for dynamic conditions in the development of a dynamic-elastic meniscal model. Novel in this study was the personalization of meniscal modeling by linking meniscal dimensions (e.g. the height and width) to the subject-specific femoral length. Starting from the scaled medial and lateral meniscus in the extended knee, the model allowed for meniscal deformation to delineate the femoral condyles during knee flexion, with the additional constraint of preserving volume. The medial and lateral menisci were modeled in the four different knee flexion configurations.

**Figure 3:**
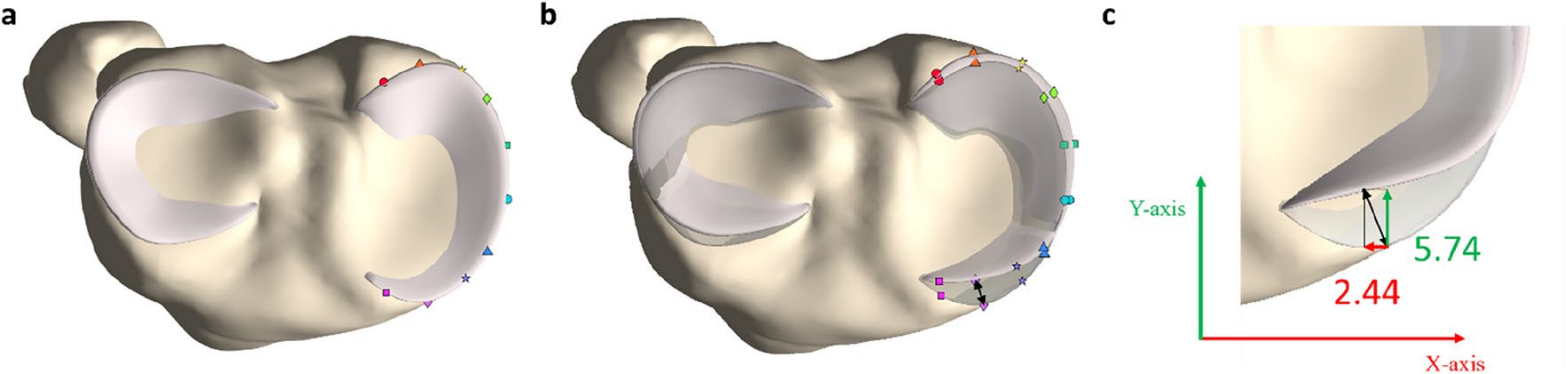
Visualization of anatomical correspondence and point-dependent distance calculations in the predicted menisci. (**a**) The medial and lateral meniscus in 0° of knee flexion were plotted in light pink on the tibial plateau (yellow). Over the outer rim of the medial meniscus, 10 points were randomly allocated. (**b**) The medial and lateral meniscus in 0° of knee flexion were plotted in light grey, including the 10 previously allocated points. Based on the principle of anatomical correspondence, the 10 corresponding points were easily identified on the medial meniscus in 30° of knee flexion as the vertices with an identical index. The black arrow indicates how the point-dependent distance was calculated. (**c**) Axial view on the anterior horn of the medial meniscus in 0° (light grey) and 60° (light pink) of knee flexion, located on the tibial plateau (yellow). Utilizing the classic approach, an anteroposterior displacement of 5.74 mm was measured (green vector). Conversely, the novel methodology computed a point-specific distance of 6.24 mm (black vector).

Furthermore, elastic deformation of the geometric morphometric model ensured the preservation of anatomical correspondence. The latter is defined as the ability to maintain the spatial relationship between corresponding anatomical structures across different poses or deformations^16^. In Fig. 3, the principle of anatomical correspondence is illustrated by manually allocating 10 points over the outer rim of the medial meniscus in 0° of knee flexion. Based on the principle of anatomical correspondence, the corresponding points were easily identified as the vertices with an identical index. This approach enabled straightforward comparison of meniscal positions across different flexion degrees. Additionally, the point-to-point distance measuring improves upon the classic antero-posterior and mediolateral measurements (Fig. 3).

### Validation of meniscal modeling

The validation of the dynamic-elastic model to predict medial and lateral meniscus anatomy inclusion in 0°, 30°, 60° and 90° of knee flexion relied on leave-one-out experiments. The theory of meniscal dynamics being dependent on tibiofemoral kinematics was tested by comparing predicted and observed meniscal geometry for the different configurations of flexion. The assessment of errors involved several metrics, including the Root Mean Square Error (RMSE), Average Surface Distance (ASD), and Hausdorff distance (HD). The RMSE represents the square root of the average of all absolute errors, the ASD indicates the average of all the absolute errors, and the HD represents the maximum absolute error. All error calculations were based on comparisons of entire meniscal meshes. Meniscal predictions were considered accurate if the average errors lied within the range of the MRI voxel size (eg. 0.3571 × 0.3571 × 1.5 mm). To identify regions with the largest prediction errors, the point-dependent errors were averaged over the different subjects and plotted.

The Shapiro–Wilk test was used to evaluate the normality assumption of the error distribution, with the alpha value set at 0.05. The null hypothesis stated that the data originated from a normally distributed population, while the alternative hypothesis stated that the data originated from a non-normally distributed population.

### Continuous dynamic meniscal modeling

#### Principal polynomial shape analysis (PPSA)

To provide a continuous description of meniscal movement between 0 and 90 degrees of knee flexion, Principal Polynomial Shape Analysis (PPSA) was employed using the approximate 0-30-60-90 degree flexion positions derived from the initial MRIs^17^. As input, PPSA utilized corresponding meshes of the femoral and tibiofibular bone in four different degrees of knee flexion, along with the corresponding, modeled medial and lateral meniscal meshes for each of the four flexion configurations.

**Figure 4:**
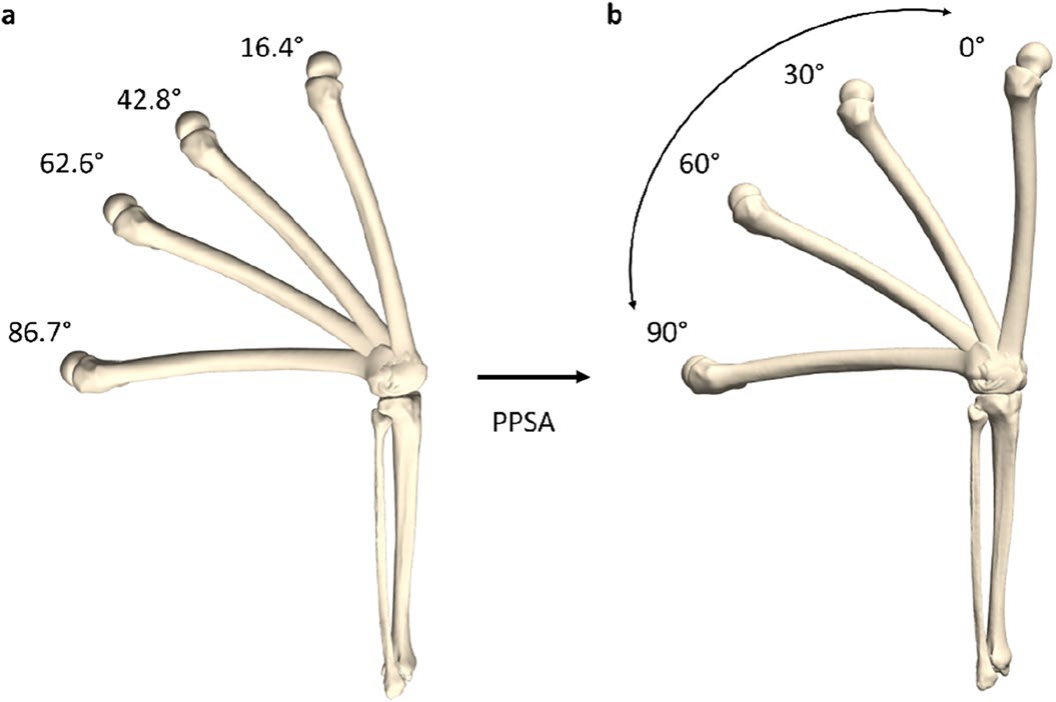
Methodological workflow to determine the position of the knee in exact 0°, 30°, 60° and 90° of flexion. (**a**) The 3D osseous configuration extracted from the MRI scans and the predicted meniscal positions in 4 different configurations of knee flexion served as the input for the PPSA model. The calculated flexion angles were added. (**b**) The development of a subject-specific PPSA model allowed to continuously describe osseous and meniscal position between 0° and 90° and to extract the exact 0°, 30°, 60° and 90° positions.

Subsequently, statistical shape analysis was performed on the articulated knee and PPSA was used subject-specifically to generate a non-linear model of leg bone geometry. By sampling the first principal polynomial, which captured the dominant variation related to flexion–extension, the knee models corresponding to the desired degree of knee flexion were selected (e.g. 0°, 30°, 60° and 90°). This allowed for reliable and accurate inter-subject comparison (Fig. 4)^21^.

#### Validation of the PPSA model

For every subject, the medial and lateral meniscal position was predicted in the same degrees of knee flexion as on the initial MRI scans, using PPSA. The PPSA-based meniscal predictions were then compared to the manually segmented menisci to assess their accuracy based on the RMSE, the ASD and the HD relying on leave-one-out experiments.

#### Description of meniscal dynamics

The subject-specific PPSA model was used to derive the exact meniscal positions at 0°, 30°, 60°, and 90° knee flexion. This allowed characterization of the meniscal movement at specific flexion angles. Next, the medial and lateral meniscus at each of the four knee flexion angles (0°, 30°, 60°, and 90°) were averaged over the 11 cases. This averaged data was then plotted to visualize the general pattern of meniscal movement across the flexion range.

Furthermore, the point-dependent distance was calculated between the 0° loaded position and the 30°, 60°, or 90° loaded positions. This distance measurement provided insights into the magnitude of displacement in different regions of the meniscus. The results were plotted to highlight the regions of the meniscus that exhibited the largest displacement during knee flexion.

**Figure 5:**
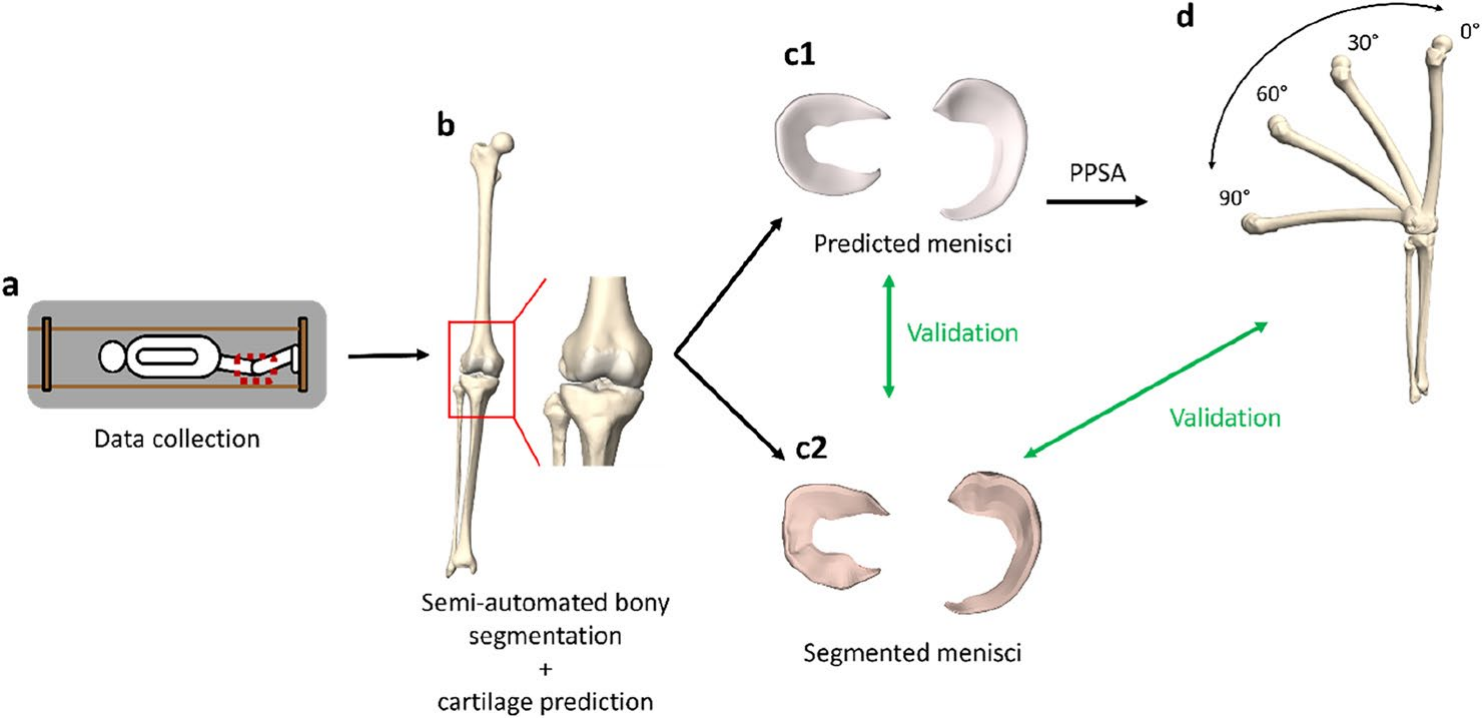
Overview of the methodological workflow. (**a**) Healthy volunteers were MRI scanned in lateral decubitus with the knee positioned in different degrees of knee flexion, approximated to be 0°, 30°, 60° and 90° of flexion using a goniometer. (**b**) Semi-automated segmentation was used to derive the osseous meshes in every bony configuration. The cartilage layer was predicted based on the methodology as described by Van Oevelen et al.^16^. (c1) The medial and lateral menisci were modeled as elastic, volume preserving meshes that delineate the femoral condyles for the 4 bony configurations. (c2) For each case and in every degree of flexion, the medial and lateral meniscus was additionally manually segmented. A comparison between the segmented and predicted meniscus was performed for validation purposes. (**d**) To continuously describe meniscal dynamics, the 4 bony configurations and the according meniscal positions served as input for subject-specific PPSA modeling. The bony and meniscal kinematics were extracted for the 4 goniometer-based flexion angles at the time of scanning to compare segmented and PPSA-predicted meniscal anatomy for validation purposes.

An overview of the complete workflow is provided in Fig. 5.

## Results

### Validation of meniscal anatomy prediction in 0°, 30°, 60° and 90°

#### Shapiro–Wilk normality test

For the various degrees of knee flexion, at least one of the variables presents a p-value smaller than 0.05 when performing a Shapiro–Wilk normality test. This indicates that the data is likely to originate from a non-normally distributed population. Consequently, the results from the different error calculations will be presented by the median and the range in the following sections.

#### Model validation in 0° of knee flexion

The prediction of the medial and lateral meniscus in the extended knee shows a similar level of error. The median RMSE equals 0.58 mm (range: 0.16–1.25) for the medial meniscus and 0.72 mm (range: 0.40–1.92) for the lateral meniscus, as shown in Table 1. These values reflect the overall accuracy of the meniscal predictions, with smaller RMSE values indicating better agreement between the predicted and manually segmented menisci.

**Table 1:** Error calculations for the course of the medial and lateral meniscus in 0° of knee flexion based on the median Root Mean Square Error (RMSE), Average Surface Distance (ASD) and Hausdorff Distance (HD) in millimeters (mm) with range.

	Medial meniscus	Lateral meniscus
RMSE (mm) (range)	0.58 (0.16–1.25)	0.72 (0.40–1.92)
ASD (mm) (range)	0.32 (0.12–0.76)	0.41 (0.18–1.36)
HD (mm) (range)	2.87 (0.60–6.00)	3.76 (1.72–6.34)

#### Model validation in 30°, 60° and 90° of knee flexion

At 30° and 60° of knee flexion, the median RMSE for both the medial and lateral meniscus ranges between 0.43 mm and 0.79 mm. However, at 90° of knee flexion, the reported median RMSE for the lateral meniscus increases slightly, reaching 1.17 mm (range: 0.32–2.08). Similarly, the median ASD and the HD increase at 90° flexion for the lateral meniscus. The median ASD equals 0.70 mm (range: 0.26–1.41) and the median HD equals 3.29 mm (range: 0.80–6.54), as presented in Table 2. These findings indicate that there is a slight increase in error in terms of meniscal position and shape prediction at 90° of knee flexion compared to the other flexion angles.

**Table 2:** Error calculations for the course of the medial and lateral meniscus in 30°, 60° and 90° of knee flexion based on the median Root Mean Square Error (RMSE), Average Surface Distance (ASD) and Hausdorff Distance (HD) in millimeters (mm) with range.

	30°		60°		90°	
	Medial meniscus	Lateral meniscus	Medial meniscus	Lateral meniscus	Medial meniscus	Lateral meniscus
RMSE (mm) (range)	0.52 (0.17–1.10)	0.79 (0.24–1.52)	0.43 (0.22–1.40)	0.60 (0.21–2.00)	0.53 (0.22–1.98)	1.17 (0.32–2.08)
ASD (mm) (range)	0.30 (0.14–0.67)	0.55 (0.20–0.97)	0.27 (0.17–0.97)	0.39 (0.16–1.55)	0.36 (0.18–1.38)	0.70 (0.26–1.41)
HD (mm) (range)	2.53 (0.47–4.16)	2.70 (0.85–6.21)	2.25 (0.28–4.42)	2.26 (1.05–5.61)	3.06 (0.89–5.63)	3.29 (0.80–6.54)

**Figure 6:**
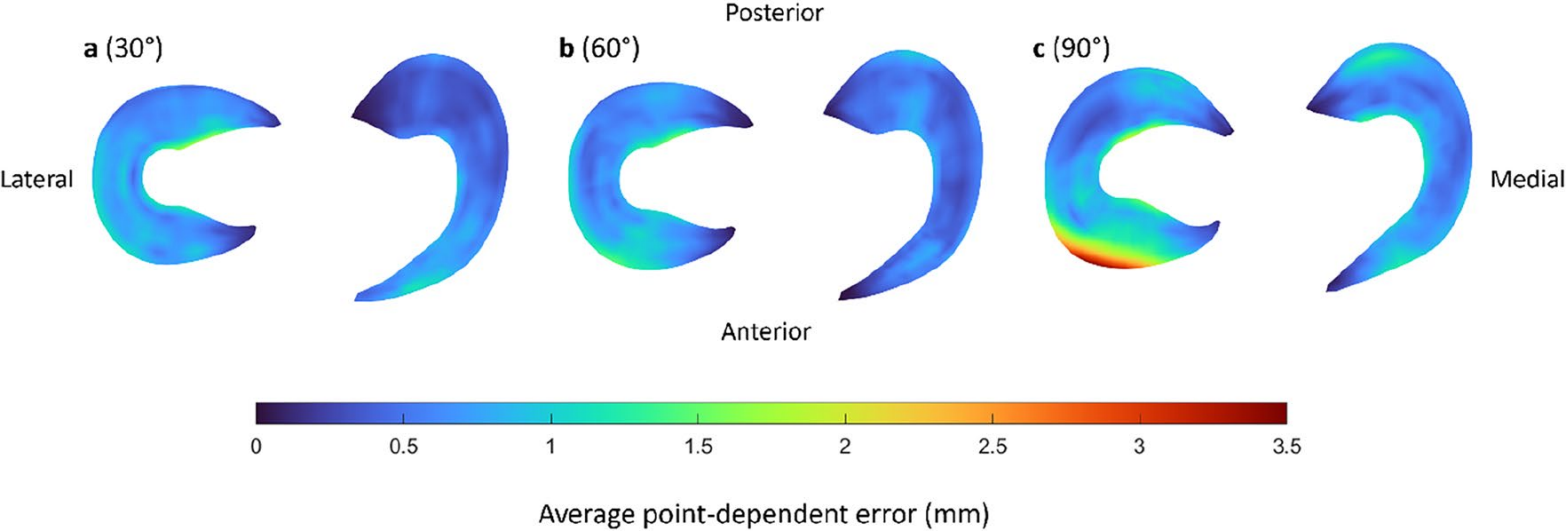
The average point-dependent error for the mean predicted medial and lateral meniscus in (**a**) 30° of knee flexion, (**b**) 60° of knee flexion and (**c**) 90° of knee flexion. The mean error is color-coded ranging from blue to red, equaling respectively an average point-dependent error of 0 mm and 3.5 mm.

Figure 6 visualizes the average point-dependent error, highlighting the regions with the largest prediction errors. The lateral meniscus shows the highest error, and this error increases with greater degrees of knee flexion. Specifically, the maximal average point-dependent error is observed at the anterior horn of the lateral meniscus in 90° of knee flexion, measuring approximately 3.47 mm (Fig. 6).

### Modeling of meniscal dynamics

#### Validation of the PPSA model

When comparing the PPSA-based predicted menisci to the manually segmented menisci, the RMSE ranges from 0.39 mm to 2.33 mm across different degrees of knee flexion. Similarly, the ASD ranges from 0.23 mm to 1.65 mm. The largest PPSA-based prediction error is located at the lateral meniscus in 90° of flexion with the median RMSE, ASD and HD equaling respectively 1.58 mm (range 0.45–2.33), 1.12 mm (range 0.34–1.65) and 4.30 mm (range 1.16–7.70) (Table 3).

**Table 3:** Comparison between the manually segmented and PPSA-based predicted medial and lateral meniscus by the median Root Mean Square Error (RMSE), Average Surface Distance (ASD) and Hausdorff Distance (HD) in millimeters (mm) with range.

	0°		30°	
	Medial meniscus	Lateral meniscus	Medial meniscus	Lateral meniscus
RMSE (mm) (range)	0.95 (0.75–1.74)	1.12 (0.50–2.29)	1.09 (0.46–2.00)	1.24 (0.53–1.98)
ASD (mm) (range)	0.72 (0.51–1.31)	0.83 (0.35–1.59)	0.77 (0.30–1.51)	0.97 (0.42–1.44)
HD (mm) (range)	3.90 (2.18–5.50)	3.41 (2.08–7.88)	3.56 (2.17–4.89)	3.15 (1.29–7.37)
	60°		90°	
	Medial meniscus	Lateral meniscus	Medial meniscus	Lateral meniscus
RMSE (mm) (range)	1.08 (0.53–1.65)	1.24 (0.58–2.05)	0.90 (0.39–1.66)	1.58 (0.45–2.33)
ASD (mm) (range)	0.77 (0.38–1.22)	0.87 (0.42–1.59)	0.64 (0.23–1.22)	1.12 (0.34–1.65)
HD (mm) (range)	3.74 (2.35–5.06)	4.15 (1.68–5.63)	3.85 (1.25–5.12)	4.30 (1.16–7.70)

#### PPSA-based description of meniscal dynamics

From 0° to 30° of knee flexion, the greatest displacement is located at the anterior horn of the medial meniscus, equaling 4.47 mm. Both the medial and lateral meniscus displace further for increasing knee flexion. A maximal displacement of 6.24 mm and 6.51 mm is measured for the mean medial and lateral meniscus respectively (Table 4).

**Table 4:** Displacement of the medial and lateral meniscus in 30°, 60° and 90° of knee flexion relative to the meniscal position in 0° of flexion reporting the maximal point-dependent displacement in millimeters (mm).

	30°		60°		90°	
	Medial meniscus	Lateral meniscus	Medial meniscus	Lateral meniscus	Medial meniscus	Lateral meniscus
Relative to zero position (mm)	4.47	3.64	6.24	5.35	5.31	6.51

**Figure 7:**
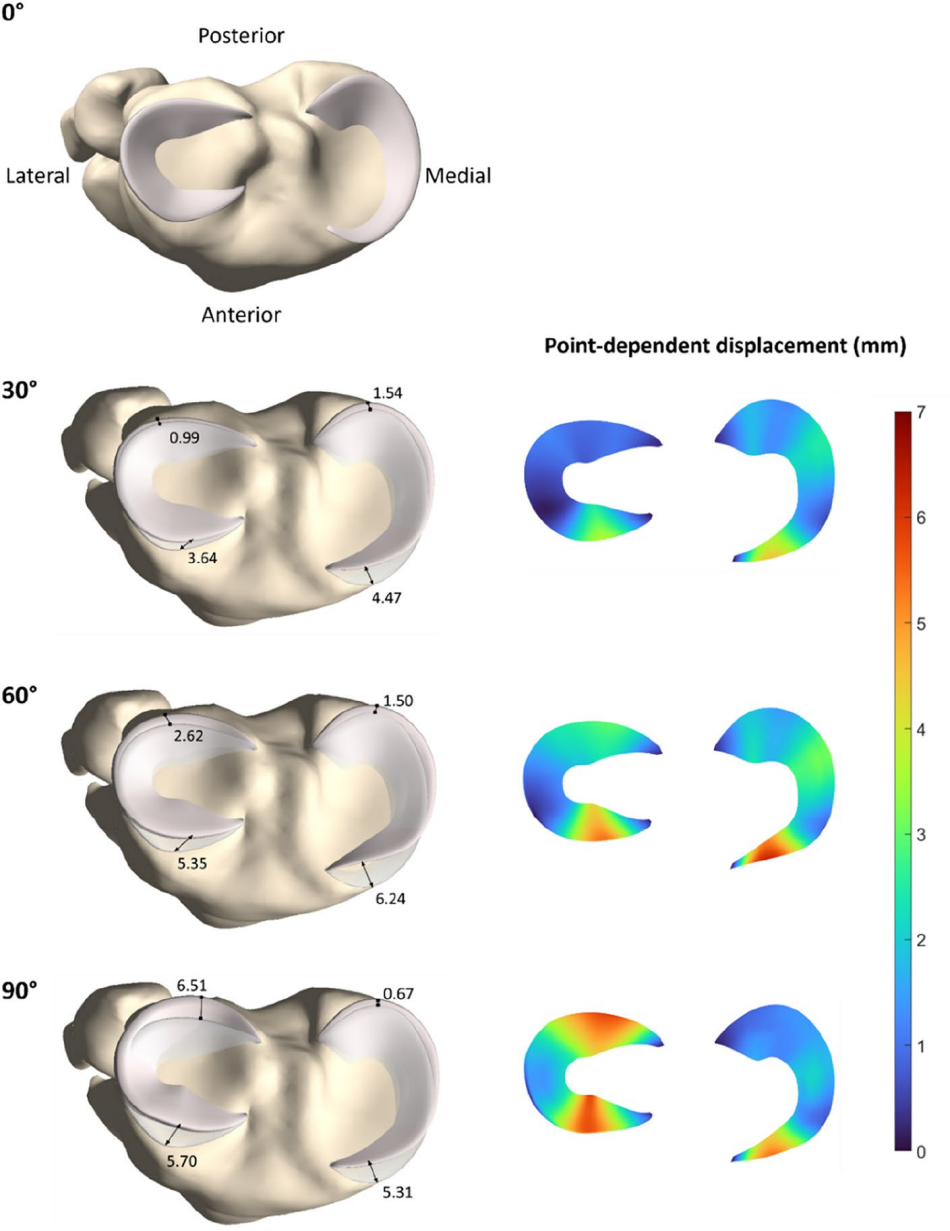
Axial view on the tibial plateau. Left: visualization of the medial and lateral meniscal position in 0° of flexion (light pink) relative to the tibial plateau (yellow). In 30°, 60° and 90° of flexion, the absolute point-dependent displacement of the medial and lateral anterior and posterior horn of the meniscus (light pink) is plotted relative to the zero position (light grey) (in mm). Right: The absolute point-dependent displacement is color-coded ranging from 0 mm (blue) to 7 mm (red).

The position of the medial and lateral meniscus is visualized relative to the tibial plateau and to the position of the menisci in 0° of knee flexion. Initially, the largest displacement is observed at the anterior horn of the medial meniscus. As the knee flexes into deep flexion, increased movement is observed in the posterior horn of the lateral meniscus, while the posterior horn of the medial meniscus remains relatively stable (Fig. 7).

## Discussion

Integrating innovative computational modeling methods, we established a meaningful relation between meniscal dynamics and the tibiofemoral kinematics at varying degrees of knee flexion. Building upon the research of Van Oevelen and colleagues, who focused on static meniscal inclusion, our novel methodologic workflow facilitates the capture of the dynamic nature of meniscal movement, employing PPSA.

Applying PPSA, we were able to model meniscal movement continuously between 0° and 90° of knee flexion and to extract the exact positions of the menisci at 0°, 30°, 60°, and 90° of knee flexion. Hence, the introduction of PPSA for continuous meniscal modeling provided accurate measurements of the meniscal positions. The extraction of menisci at exact and defined flexion angles using PPSA overcame the limitations of goniometer-controlled MRI scans and facilitated more accurate comparisons of meniscal positions between subjects. This computational approach enables us to obtain a comprehensive understanding of how the menisci move and adapt throughout the range of knee flexion. Beyond enabling personalized and accurate prediction of meniscal position during knee flexion, averaging positions across multiple subjects provides valuable insights into meniscal dynamics on a population-level.

Through the utilization of these innovative computational techniques, we verified a close relationship between meniscal dynamics and tibiofemoral kinematics. This highlights the significance of tibiofemoral movement analysis for improved understanding of meniscal kinematics^22^. In vivo analysis of tibiofemoral motion in the sagittal plane during the stance phase of gait reveals specific patterns. Initially, at heel strike, the knee is on average 5° of knee flexion^23–25^. During the loading response, there is a slight increase of flexion, followed by extension during midstance. At terminal stance, flexion is initiated, reaching a maximum of approximately 36°. Many researchers report a distinction in the displacement patterns of the medial and lateral condyles during initial knee flexion. While the lateral condyle exhibits a more pronounced displacement, the medial condyle demonstrates minimal movement, which is known as medial-pivoting. Hill and colleagues described an extension and a flexion facet on the tibial plateau. The extension facet comprises an uphill slope of approximately 11°. Between 10° and 30° of knee flexion, the medial femoral condyle rocks between the extension and the flexion facet. This articulatory motion does not involve substantial displacement of the condyle, but rather a posterior shift in the contact area between the condyle and the tibia^26^. Similarly, the findings of Postolka et al. confirmed a medial pivot during unloaded knee flexion^27^.

However, growing evidence suggests increased medial condylar movement, particularly in the anteroposterior direction, during initial knee flexion under dynamic gait conditions^24,28^. Grieco et al. observed medial condylar posterior displacement of 4.7 mm between 0 and 30° of knee flexion, measured during gait and utilizing mobile fluoroscopy^29^. Additionally, Kozanek et al. investigated condylar motion during the stance phase of treadmill gait utilizing dual fluoroscopy imaging. They observed greater excursions of the medial femoral condyle in the anteroposterior direction relative to displacement of the lateral femoral condyle^24^. Consistent with these findings, our study reports the largest displacement in the range between 0° and 30° of knee flexion to be located at the anterior horn of the medial meniscus. In contrast, Liu et al. reported larger movements in the lateral meniscus in 30° of flexion. However, it is important to note that they defined meniscal displacement as the movement of the medial and lateral centroids relative to the plateau, which may have influenced their findings^11^.

As knee flexion increases, a phenomenon known as femoral rollback is observed, wherein the femoral bone moves posteriorly relative to the tibial plateau^13,28,30^. Hill et al., Pinskerova et al. and Victor et al. reported that the lateral femoral condyle exhibits a greater excursion than the medial condyle at higher ranges of flexion^26,30–32^. Similarly, Johal et al. described increased lateral femoral condyle movement for deep squatting^33^. With meniscal dynamics following tibiofemoral kinematics, large lateral meniscal displacement is to be expected.

In line with the observed tibiofemoral kinematics, our study found increased lateral meniscal movement with increasing knee flexion. These findings are consistent with previous studies. Thompson et al. reported the largest displacement to be located at the anterior horn of the lateral meniscus, measuring 12.8 mm for complete knee flexion. They also reported a mean displacement of 9.6 mm for the lateral posterior horn. In comparison, the medial meniscus exhibited lesser movement, with anterior and posterior horn displacements measuring 7.0 mm and 3.9 mm, respectively^2^. Similarly, De Coninck et al. reported lateral meniscal displacement of 11.2 mm and 7.7 mm for respectively the anterior and posterior horn^15^. Regarding the medial meniscus, both Thompson et al. and De Coninck et al. reported reduced posteromedial mobility, with absolute displacement values measuring 3.2 mm and 5.0 mm respectively. Alike, our study also identified decreased mobility in the medial posterior horn, contributing to posteromedial joint stability.

These findings collectively highlight the differential movement patterns of the medial and lateral menisci during knee flexion. The greater mobility observed in the lateral meniscus, particularly in the posterior horn, corresponds to the kinematic behavior of the lateral femoral condyle. Conversely, the medial meniscus exhibits more restricted movement, emphasizing its role in providing stability and preventing excessive anterior tibial translation during deep knee flexion, functioning as a secondary stabilizer^2,5,28^.

The results of the leave-one-out validation experiments provide additional support to our findings concerning meniscal dynamics. When comparing error calculations for meniscal inclusion in the extended knee, we observed slightly smaller RMSE and ASD values compared to a similar study by our group^16^. This improvement in meniscal modeling can be attributed to the incorporation of a subject-specific scaling factor, based on the femoral length. Additionally, we found similar errors in meniscal modeling in 30°, 60° and 90° of knee flexion. The ASD errors were in same order of magnitude as the MRI voxel size, indicating that the prediction errors are comparable to the errors associated with manual segmentation, which is commonly regarded as the ground truth. These findings allowed us to validate the accuracy of meniscal inclusion in different degrees of knee flexion, supporting the theory that the menisci closely follow the geometry and position of the femoral condyles relative to the tibial plateau. The results further reinforce the notion that meniscal dynamics are intricately linked to the tibiofemoral kinematics observed during knee flexion.

However, there are variations in the absolute values of meniscal displacements reported among different studies. In comparison to the findings of Thompson and colleagues, our study as well as others have reported smaller meniscal displacements^2^. Vedi et al. reported smaller lateral meniscal displacements of 9.5 mm and 5.6 mm for the anterior and posterior horn respectively in 90° of flexion^5^. Kim et al. observed a lateral anterior and posterior horn displacement of 6.61 mm and 7.53 mm respectively^8^. These discrepancies can be attributed to various factors. One potential explanation for the reduced meniscal mobility observed in our study and other in vivo studies is the presence of a viable soft tissue envelope surrounding the knee joint. This soft tissue envelope acts as a restraint and can limit the range of motion and displacements of the menisci^2,5,33^.

Even within studies comparing subjects with a viable soft tissue envelope, measured differences in meniscal displacement can be attributed to the various methodologies employed. The lack of a consensus on how to evaluate meniscal displacement complicates the direct comparison of absolute displacement values reported by different research groups. Different methodologies have been used, including measuring anteroposterior meniscal displacement relative to the tibial plateau on a single MRI slice, evaluating the displacement of a single point such as the tibiofemoral contact area centroid, or employing anatomical landmark matching algorithms to register the positions of manually segmented menisci. Vedi et al. and Kim et al. measured anteroposterior meniscal displacement relative to the tibial plateau on a single MRI slice^2,5,8^. Shefelbine et al. assessed meniscal movement by evaluating the displacement of just one single point, namely the tibiofemoral contact area centroid^13^. Ma and colleagues introduced an anatomical landmark matching algorithm to register the tibial bones and compare the positions of manually segmented menisci. However assessed on three-dimensional menisci, once more solely the anteroposterior meniscal movement relative to the tibial plateau was measured^6^. These variations in measurement techniques and reference points make it challenging to directly compare absolute displacement values across studies. To address these challenges and improve the understanding of meniscal displacement, it is important to establish standardized methodologies for evaluating meniscal movement and to consider multiple factors, including three-dimensional displacements and the influence of the soft tissue envelope, in future research.

Introducing point-dependent, three-dimensional meniscal displacement calculations based on anatomical correspondence is a significant advancement in our study. This methodology, integrated within the statistical shape modeling-based workflow, allows for precise measurement of meniscal displacement at specific points across different degrees of knee flexion. By calculating the Euclidean distance between corresponding meniscal points in different flexion angles, we can determine the magnitude and direction of displacement in three-dimensional space. This approach enables us to identify specific meniscal zones that experience tension along the longitudinal fibers of the tissue, providing valuable insights into the complex three-dimensional deformations of the meniscus. By considering meniscal deformation rather than just displacement in a single plane, we gain a more comprehensive understanding of how the meniscus adapts and moves during knee flexion. In addition, the use of point-dependent distance calculations allows for automatic and standardized measurements, eliminating potential errors and subjectivity associated with manual measurements. This enhances the reliability and reproducibility of our findings and facilitates comparisons across different subjects and studies.

### Strengths

Our methodological workflow offers several advancements in the study of meniscal dynamics. By combining personalized meniscal inclusion, PPSA-based modeling, and the concept of anatomical correspondence, we have achieved a comprehensive understanding of meniscal movement in different degrees of knee flexion. This approach allows for continuous assessment of meniscal dynamics without the need for repeated MRI scans and manual segmentation.

The inclusion of personalized meniscal models based on subject-specific data enhances the accuracy and relevance of our findings. Moreover, by extracting the exact meniscal position in any degree of flexion, we ensure standardized comparisons between multiple cases. This enables us to analyze both subject-specific and general meniscal dynamics.

The utilization of a 3 Tesla MRI provides high-resolution imaging and allows for visualization of meniscal details even during deep knee flexion. While our study focused on subjects shorter than 1.70 m, subject-specific modeling in individuals of varying heights remains viable as we implemented a subject-specific scaling factor derived from femoral length.

By introducing the concept of anatomical correspondence in meniscal displacement calculations, we have advanced the understanding of three-dimensional meniscal deformations. This approach provides insights into mediolateral displacements and tensioning along the longitudinal fibers of the meniscus, going beyond traditional anteroposterior measurements.

In summary, our methodological workflow offers a comprehensive and innovative approach to studying meniscal dynamics. It has the potential to enhance our understanding of the role of the meniscus in knee function and contribute to the development of improved diagnostic and treatment strategies for meniscal pathologies.

### Limitations

It is important to acknowledge the limitations of our study and consider the implications for generalization and further research. The included subjects in our study were all of western European descent, which limits the generalizability of our findings to other populations. The complex interplay between genes, environment, and culture can result in population-based variations in morphological features. Therefore, caution should be exercised when extrapolating our results to individuals from different ethnic backgrounds.

Furthermore, our model development was based on a relatively small sample size of 11 healthy volunteers. The scanning was performed with the subject positioned sideways, simulating weight-bearing conditions by applying an additional load equivalent to approximately half of their body weight. Disparities will exist when comparing the scanned tibiofemoral kinematics to the effective tibiofemoral kinematics observed during gait. Nevertheless, this does not cast doubt on the concept of meniscal dynamics being interconnected with tibiofemoral kinematics.

Lastly, it is important to recognize that statistical modeling of soft tissue, including the menisci, is an approximation of the true anatomical reality. Balancing the inclusion of sufficient anatomical detail with computational efficiency requires certain simplifications in our modeling approach. However, the validation experiments conducted in our study demonstrated that the included anatomical detail was sufficient, as the prediction errors were in the same order of magnitude as the MRI resolution.

### Future directions

Moving forward, it will be valuable to conduct larger-scale studies involving diverse populations and pathological conditions to further validate and refine our understanding of meniscal dynamics. While the acquired dataset allows us to investigate meniscal dynamics in the context of a healthy knee joint, it may not capture the complexities and variations present in pathological conditions. The influence of specific pathologies, such as ACL deficiencies, on three-dimensional meniscal dynamics is still uncertain. It is known that ACL-deficient knees exhibit altered tibiofemoral kinematics, which may impact meniscal movement. For example, Shefelbine and colleagues concluded that tibiofemoral kinematics are altered in the ACL—deficient knees^13^. However, further research is needed to validate and explore the relationship between pathology and meniscal dynamics.

Additionally, advancements in imaging technology and modeling techniques hold immense promise for the future. Specifically, the emergence of technologies enabling fully dynamic measurements of meniscal movement could further improve upon our understanding of menisci in various clinical scenarios.

## Conclusion

Through the utilization of an advanced computational modeling workflow, we confirmed that meniscal dynamics are strongly linked to tibiofemoral kinematics. Consistent with earlier research, our results verify that the most substantial meniscal displacements occur at the anterior horn of the medial meniscus during the initial knee flexion. In deep knee flexion, we observed the most significant meniscal displacement to be located at the posterior horn of the lateral meniscus. Furthermore, the observed relative stability of the posterior horn of the medial meniscus in deep knee flexion plays a role in enhancing posteromedial joint stability.

Combining personalized meniscal inclusion and PPSA-based modeling, a more reliable and standardized approach for studying the complex interaction between the menisci and knee joint kinematics is provided. Additionally, the introduction of point-dependent distance calculations based on anatomical correspondence enables automatic and standardized distance calculations, surpassing manual measurements.

In summary, our study successfully demonstrates a strong correlation between meniscal dynamics and tibiofemoral kinematics, achieved through the implementation of innovative computational techniques.

## Data Availability

The data analyzed in this study is subject to the following licenses/restrictions: Digital bony shapes of the lower limb in different positions of flexion were extracted from a database of 12 healthy volunteers (6 females and 6 males). Requests for data access should be directed to the corresponding author (emmanuel.audenaert@ugent.be).
